# Genome-wide analysis of alternative splicing in cow: implications in bovine as a model for human diseases

**DOI:** 10.1186/1471-2164-10-S3-S11

**Published:** 2009-12-03

**Authors:** Elsa Chacko, Shoba Ranganathan

**Affiliations:** 1 Department of Chemistry and Biomolecular Sciences and ARC Centre of Excellence in Bioinformatics, Macquarie University, Sydney, NSW 2109, Australia; 2 Department of Biochemistry, Yong Loo Lin School of Medicine, National University of Singapore, 8 Medical Drive, Singapore 117597

## Abstract

**Background:**

Alternative splicing (AS) is a primary mechanism of functional regulation in the human genome, with 60% to 80% of human genes being alternatively spliced. As part of the bovine genome annotation team, we have analysed 4567 bovine AS genes, compared to 16715 human and 16491 mouse AS genes, along with Gene Ontology (GO) analysis. We also analysed the two most important events, cassette exons and intron retention in 94 human disease genes and mapped them to the bovine orthologous genes. Of the 94 human inherited disease genes, a protein domain analysis was carried out for the transcript sequences of 12 human genes that have orthologous genes and have been characterised in cow.

**Results:**

Of the 21,755 bovine genes, 4,567 genes (21%) are alternatively spliced, compared to 16,715 (68%) in human and 16,491 (57%) in mouse. Gene-level analysis of the orthologous set suggested that bovine genes show fewer AS events compared to human and mouse genes. A detailed examination of cassette exons across human and cow for 94 human disease genes, suggested that a majority of cassette exons in human were present and constitutive in bovine as opposed to intron retention which exhibited 50% of the exons as present and 50% as absent in cow. We observed that AS plays a major role in disease implications in human through manipulations of essential/functional protein domains. It was also evident that majority of these 12 genes had conservation of all essential domains in their bovine orthologous counterpart, for these human diseases.

**Conclusion:**

While alternative splicing has the potential to create many mRNA isoforms from a single gene, in cow the majority of genes generate two to three isoforms, compared to six in human and four in mouse. Our analyses demonstrated that a smaller number of bovine genes show greater transcript diversity. GO definitions for bovine AS genes provided 38% more functional information than currently available in the sequence database. Our protein domain analysis helped us verify the suitability of using bovine as a model for human diseases and also recognize the contribution of AS towards the disease phenotypes.

## Background

Protein diversity in eukaryotic genomes is mainly credited to alternative splicing (AS). It is a fundamental mechanism by which a single pre-mRNA can produce more than one transcript. It is also considered by many to be an important mechanism for controlling gene expression [1]. The introns in the pre-mRNA are spliced out and the exons are united in different combinations leading to a change in the primary transcript structure. This change in transcript structure can affect the encoded protein thereby disrupting its structure and also its function. The disruption in the protein structure and function brought about by AS are frequently associated with diseases [2]. Results from previous studies indicate that more than 60% of human genes are alternatively spliced [3-9].

Association of AS with many diseases such as cardiovascular, cancer and neurodegenerative disorders sheds light on the fact that it is crucial to conduct an in-depth study on AS [10]. Analyses have also shown that 15% of point mutations that cause genetic disease affect pre-mRNA splicing [10], providing a link between AS events and inherited genetic diseases.

Large scale sequencing of eukaryotic genomes and the knowledge of AS being an important player in controlling gene regulation has seen the emergence of several efforts [3-9] to create bioinformatics resources on alternative transcripts and protein isoforms [11]. Conflicting results from previous analyses aiming to compare the rate of alternative splicing between different organisms contradict AS databases who discuss genome-wide computational analysis. All vertebrates and invertebrates showed a similar rate of alternative splicing with respect to both the number of genes affected and the number of variants per gene in a large-scale expressed sequence tag (EST) analysis across distinct eukaryotes by Brett and coworkers [12]. On the contrary considerable variation in the rates of alternative splicing across organisms was reported by Lee and co-workers [5]. Understanding the phenomenon of AS is difficult as these databases do not provide sufficient information for multi-gene comparison across various species. ASAP II [5] concentrates mainly on comparative and evolutionary studies. ECGene [9] provides functional annotation for AS genes in various genomes. Alternative Splicing Transcript Database (ASTD) [3,4] does an exhaustive analysis of AS events in three species, namely human, mouse and rat. Representing the transcripts and their relation to each other has become extremely complicated due to the increasing number of transcripts for each gene. This has seen the dawn graph theory and its application to represent a gene transcript. Graph theory is a prominent concept that has been used to express transcripts and capture their relation, among many other solutions. The language of graph theory offers a mathematical abstraction for the description of biological relationships [13]. Modrek and Lee used directed acyclic graphs for EST analysis, with the genomic DNA sequence as reference [14]. Pevzner and coworkers [15] were the first to use de Bruijn graphs to depict the transcripts alone, without referring to the genomic DNA sequence, where the maximum common sub-sequences between transcripts were condensed into nodes and the variable regions connected by edges. Alternative Splicing Gallery (ASG) resource uses such an approach [7].

Our group has used directed acyclic splicing graphs, without a genomic DNA sequence as reference, with exons as nodes, interconnected by introns as edges, where the paths through the splicing graph represents the transcripts. This scheme was applied to the genome-wide analysis of *Drosophila melanogaster *[6], leading to the DEDB data resource. Here, the first transcript served as a reference sequence to generate splicing graphs, with automatic rule-based classification of splicing events. To reduce the uncertainty in selecting the primary transcript, this methodology was further enhanced. The most conserved exons in all transcripts of a given gene were chosen to be distinct reference exons and all others were considered to be variant exons. In order to generate a splicing graph from a set of transcripts for a given gene, we thereby developed the Alternative Splicing Graph Server (ASGS) [8].

As a part of the bovine genome annotation team, we have used comparative genomics in order to associate alternative splicing patterns in human and mouse to cow [16]. Comparative genomics studies the correlation between genome structures and functions across different biological species. It aims at understanding many aspects of the evolution of modern species.

The intermediate evolutionary distance between human and bovine is 70-100 Myr [17]. The bovine model has been found to be relevant to human health research priorities such as obesity, female health and communicable diseases. Cow provides a valuable biological model in these significant areas because of the vast amount of research that has been conducted with respect to genetic and environmental interactions associated with complex, multi-genic physiological traits [18]. The Cetartiodactyl order of mammals, to which cattle and all other ruminants belong, is phylogenetically distant from the primates, and thus contains invaluable information for understanding human genome evolution [19].

In this study, we have analysed transcripts for each gene in the bovine genome. Since the bovine genome is not yet completely annotated we minimized any gene structure bias in the input data by carrying out comparative genome analysis on the orthologous subset of AS genes for the three species. We present here the comprehensive analysis of all bovine, human and mouse transcripts based on splicing graphs. AS events in these three genomes and their functional significance in terms of gene ontology (GO) [20] classifications were also identified. The two main AS events (cassette exons and intron retention) in the human disease genes (94) from NCBI Genes and Disease database [21] were mapped onto their respective bovine orthologous genes. A protein domain analysis on 12 human disease genes that are known to be occurring in cow was vital in providing significant insights into the protein structure/function affects of AS.

## Materials and methods

### Data

For AS analysis, the GTF files for *Bos taurus, Homo sapiens *and *Mus musculus *were extracted from Ensembl ver. 54 [22]. Each line in the Gene Transfer Format (GTF) [23] file corresponds to the structure of the exons making up the transcripts, coding sequence, start codon and stop codon information. For our analysis, we extracted only the protein coding genes and eliminated the pseudo genes and mitochondrial genes. The unspliced transcript sequences were also obtained from Ensembl for cow to analyse the splice site motifs.

### Splicing graphs

The procedure used in ASGS [8] has been adopted for compiling the graphs. The transcript information, including start and stop of each exon are compiled from the GTF file for each of the three genomes to generate the splicing graph. All transcripts are converted to the leading strand for consistency. Exons are divided into two main groups; distinct and variant. The exon that occurs in the majority of transcripts is retained as the distinct exon, with the rest classified as variant. When exons overlap, the exons with well-determined borders, occurring in most of the transcripts is considered to be distinct. If an exon is completely contained in another larger exon, these are not merged but retained as individual exons, considered variant and then entered into a list maintaining the mapping of variant exons to distinct exons [24]. Splicing graphs are then generated using these distinct and variant exons. The first line of the resultant splicing graph is composed entirely of distinct exons, followed by subsequent lines showing the locations of variable exons. The exons are connected by edges, representing introns in the set of transcripts provided. Splicing graphs were compiled for every alternatively spliced gene for the three genomes. The splicing graphs were then further analysed to identify the splicing events and patterns for orthologous genes.

### Detection and classification of alternative splicing events and patterns

**Figure 1:**
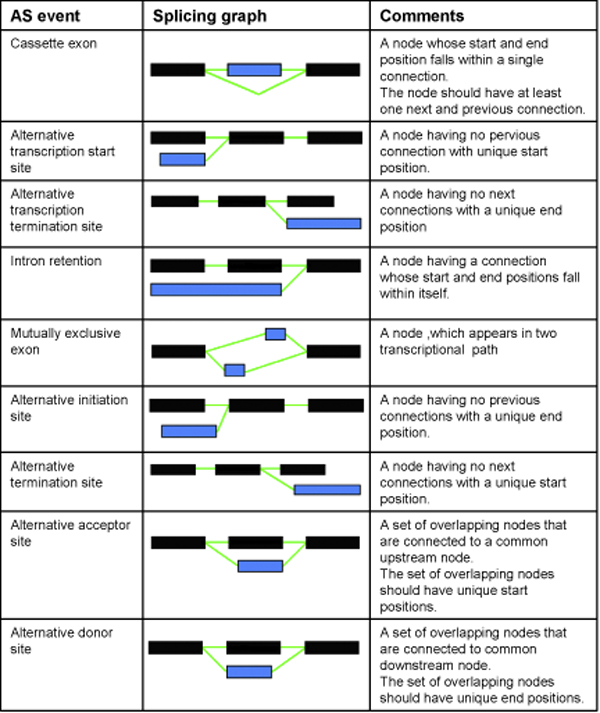
**Generation of alternative splicing (AS) events using splicing patterns**. Rules were derived to detect nine alternative splicing events. Distinct exons are shown in black, while variant exons are shown in blue.

We have analysed nine alternative splicing events namely, cassette exons, intron retention, alternative donor sites, alternative acceptor sites, alternative transcriptional start and termination sites, alternative initiation and termination exons and mutually exclusive exons. Figure 1 defines the rules to locate each of the nine events and these rules were applied to generate the splicing graphs. This classification schema has been previously described in DEDB [6] and incorporated into ASGS [8] for the identification of the splicing events. 5' and 3' ends of the transcripts are usually difficult to determine experimentally due to sequencing errors which could cause anomalies in the analysis of alternative transcriptional start and termination sites [6]. The other internal AS events, however, are not affected by these sequencing errors. Two types of analyses namely gene level and event level were carried out. The percentage of total events present in each genome for the orthologous genes is portrayed by the event level analysis. The gene level analysis calculates the percentage of all AS genes and orthologous AS genes showing each of the events for the three genomes.

**Figure 2:**
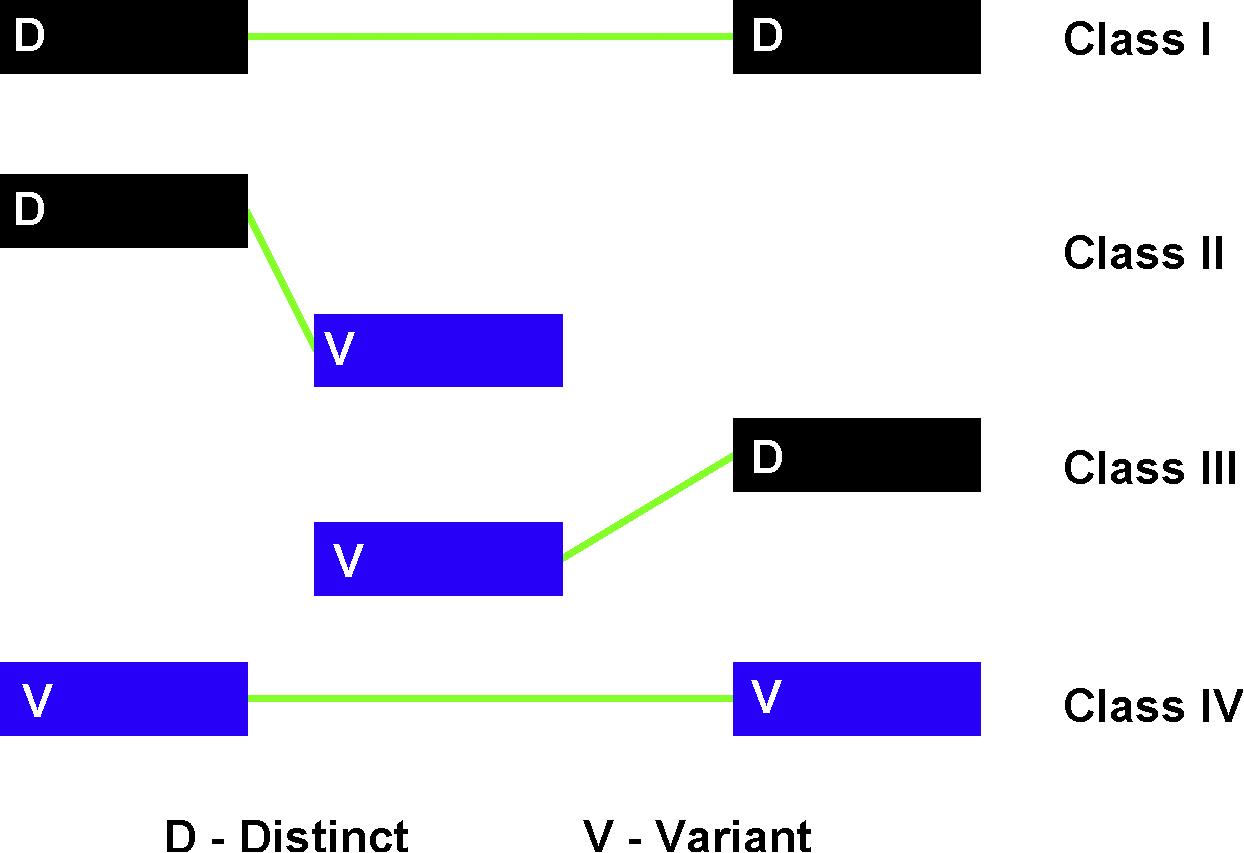
**Classification of inter-exonic connections as splicing patterns**. Four component splicing patterns have been defined, depending on connections between distinct exons (black) and variant exons (blue). Class I refers to connections between two successive distinct exons while Class IV refers to connections between two successive variant exons. Classes II and III depict connections between a distinct exon and a variant exon and vice-versa.

Splicing graphs have been made more informative to help identify distinct and associated variant exons by visual representation of distinct (D) exons in black and variant (V) exons as blue. AS events can therefore be depicted using a minimum of four sub-graph components called splicing patterns. Figure 2 depicts the four unique sub-graphs Class I (D-D), Class II (D-V), Class III (V-D) and Class IV (V-V). The fundamental definition of transcript diversity is given by a detailed analysis of the relationship of each exon to its successor, designated as a splicing pattern.

### Qualitative and quantitative analysis of exons and introns

Basic statistical measures like the mean, median and standard deviation were calculated for all three genomes in order to analyse the exon and intron size conservation across the three genomes for the complete and orthologous AS gene sets. The number of exons per transcript for the three genomes was also calculated.

### Splice site motif analysis

Splice site mutations are believed to cause several genetic diseases. It is therefore very important to identify variations in the splice site. The frequencies of GT-AG, GC-AG, AT-AC splice site motifs were computed for bovine and analysed and compared to the splice site information for human and mouse obtained from ASTD.

### GO annotation

Analysis of the GO annotations was conducted for two sets of data. In the first set, the transcript sequences of orthologous bovine AS genes obtained from Ensembl were processed using ESTScan, as it can detect and extract coding regions from low quality sequences with high selectivity and sensitivity and is also able to accurately correct frame shift errors [25]. To obtain even datasets, the human and mouse transcript sequences were also processed using ESTScan. The output was then processed using another bioinformatics tool, Blast2GO [26], which we have successfully used in the annotation of expressed sequence tag sequences [27]. The BLAST results from this program were then mapped to GO terms to obtain the GO annotation. The annotation output file was then processed using a plotting tool, WEGO [28] in tool to compile the GO annotation results into category-based lists.

The second dataset was a text file comprising GO annotations for bovine AS genes orthologous to human and mouse AS genes, obtained from Ensembl using the BioMart [29] tool. The second dataset was reformatted and put through the WEGO tool to compile the GO annotation results for plotting.

### Mapping of human disease genes to bovine orthologous genes

A well-annotated set of all available (94) human disease genes was extracted from NCBI Genes and Disease database [21], with the view towards analysing which of these genes were alternatively spliced in human and bovine genomes. Of these 94 genes, AS analysis was conducted on the 66 spliced genes (with more than one transcript). The two most important events, cassette exon and intron retention, were examined in detail in these 66 genes. These exons were then mapped onto the orthologous exons in bovine using CLUSTALX [30] multiple sequence alignment tool to identify the conservation of these exons and the splicing event, across the two species. Irrespective of the position of the exons in different transcripts, if two pairs of exons have a good percentage of alignment they are still considered as conserved exons, thereby implying that in the event of exon shuffling, the exon pairs are still considered conserved.

### Protein domain analysis of the orthologous disease gene set

We identified eight human disease genes that have bovine orthologues. The protein sequences encoded by the transcripts for these human and bovine genes were analyzed using Pfam [31] domain search tool to identify the effects of alternative splicing on the functional protein domains.

## Results and discussion

It was observed that only 21% of bovine genes were alternatively spliced as opposed to 68% of genes in human and 57% of genes in mouse upon comparison of 4567 bovine AS genes with 16715 human AS genes and 16491 mouse AS genes. The statistics provided by ASAP II database (26%, 53%, 53% for cow, mouse and human respectively) [5] compare well to these estimates of the number of AS genes in cow, mouse and human, although they appear almost twice as much as those reported by Nagasaki and group [32] (32.1% and 23% for human and mouse genomes, respectively). All AS genes in cow which have alternatively spliced orthologues in both human and mouse were extracted to minimize any gene structure bias and to get the best-annotated genes in cow for analysis. Such an approach has been adopted by the studies of Chen et al [33]. In order to compile the orthologous genes subset, one-to-one, many-to-many, one-to-many and apparent mappings have been used. We found that 3504 genes in cow have alternatively spliced orthologues in human and mouse amounting to 3835 and 3774 genes respectively. This dataset amounted to 16% of bovine alternatively spliced genes, compared to 16% in human and 13% in mouse. Our values are consistent with those (10%) observed by Brett et al. [12] for AS between human and other species, including mouse and cow reinstating the credibility of our approach of using orthologous AS gene subsets for multi-species comparisons and to estimate the extent of AS in cow.

### Qualitative and quantitative analysis of exons and introns

Compared to 8.0 and 6.5 transcripts per gene in human and mouse respectively, our results indicate that bovine AS genes are represented by 2.3 transcripts per gene on average. Overall, bovine AS genes show less transcript diversity compared to human and mouse AS genes as indicated by these numbers which are quite similar to those in the orthologous gene set as well. General statistical characteristics of the intron-exon structure of eukaryotic genomes are invaluable for understanding the structure and evolution of genes and genomes. Deutsch and Long [34] estimated that each gene comprises 5.0 exons of mean length 51 nt separated by introns of mean length 3413 nt; and 4.4 exons of mean length 52 nt separated by introns of mean length 1321 nt for human and mouse genes, respectively, using available gene structure information on ten model organisms. We found that each bovine transcript comprises close to 13 exons of mean length 181 nt, separated by introns of mean length 5215 nt, while human and mouse transcripts comprise close to 8 and 7 exons, respectively, of mean length 178 and 160 nt, respectively; separated by introns averaging 5314 and 4311 nt, respectively (Table 1). While all three transcriptomes are composed of exons and introns of similar size, bovine AS genes are more fragmented than human and mouse AS genes since these numbers are again similar to those obtained for the orthologous AS gene set.

**Table 1:** Comparison of alternative splicing in bovine, human and mouse genomes

Genome	Genes	Genes with multiples transcripts	% of alternative splicing	Transcripts per gene (mean ± sd (med))	Exon numbers per transcript (mean ± sd (med))	Exon size (nt) (mean ± sd (med))	Intron size (nt) (mean ± sd (med))
Bovine	21755	4567	21%	2.3 ± 1.2 (3)	13.4 ± 10.5 (9)	181 ± 254 (126)	5215 ± 17003 (1191)
Human	24573	16715	68%	8.0 ± 8.0 (7)	7.7 ± 5.9 (6)	178 ± 196 (89)	5314 ± 4112 (4517)
Mouse	28931	16491	57%	6.5 ± 6.0 (5)	6.6 ± 4.2 (5)	160 ± 167 (63)	4311 ± 4003 (3889)

**Orthologous gene set**							

Bovine	21755	3504	16%	2.5 ± 1.0 (3)	14.4 ± 9.8 (10)	162 ± 212 (123)	5105 ± 16900 (1152)
Human	24573	3835	16%	9.4 ± 7.4 (8)	9.1 ± 7.8 (8)	188 ± 150 (101)	5210 ± 4013 (4321)
Mouse	28931	3774	13%	7.0 ± 6.4 (6)	9.0 ± 7.2 (7)	145 ± 163 (103)	4304 ± 3921 (3789)

Mean, standard deviation (sd) and median (med) values have been computed for columns 5-8.

### Splicing graphs

**Figure 3:**
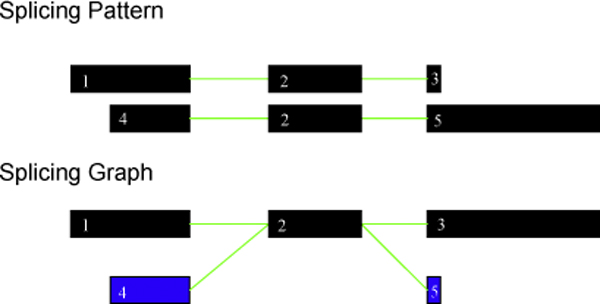
**Splicing graph for the human disease gene Myc (Burkitt Lymphoma)**. The splicing graph represents the gene in a very simple and easily understandable format.

We generated a total of 4567 bovine, 16715 human and 16491 mouse splicing graphs. The transcript structure of each multi-transcript gene for all three genomes was compiled using the splicing graph approach [8]. The splicing graphs were further decomposed into component splicing patterns (as described in Materials and methods). It was noted that 2485 bovine genes are single exonic genes. It is possible to verify all the splicing events from the splicing graphs thereby suggesting that it could be utilised as an excellent visual analysis tool. One such splicing graph of *Myc *responsible for causing the disease Burkitt Lymphoma is shown in Figure 3. It can be easily seen from Figure 3 that the gene has two different transcripts.

### Alternative splicing events and patterns

The nine AS events discussed above have been identified in the orthologous set for bovine genome and are compared to those in human and mouse. Equation 1 was used to calculate the % of genes showing each AS event in each of the three genomes for the gene level analysis.

**Figure 4:**
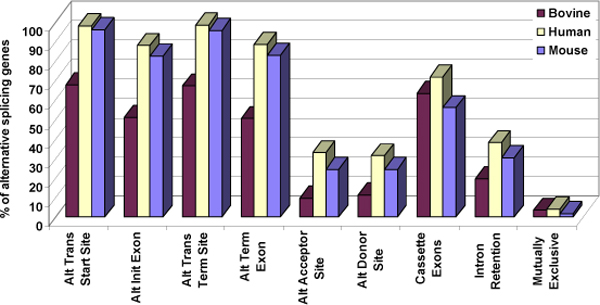
**Distribution of AS events - gene level analysis for bovine, human and mouse orthologous AS genes**. Nine events, described in Figure 1, were used to classify the observed AS phenomena based on the number of genes displaying these events, as shown in Table 2.

The first four AS event categories in Figure. 4, refer to splicing events at the ends of a gene, while the remaining five represent internal events. The results of our gene level analysis highlight that most of the genes showed external events. As suggested earlier the high percentage for transcriptional start and termination sites events could be the result of sequencing errors. We observed that majority of the genes in cow (59%-64%) have cassette exons, with 19%-20% of the genes having intron retention. Very few genes exhibited mutually exclusive exons (3%-4%). Figure 4 clearly shows that fewer bovine genes exhibit AS events than that of those in human and mouse. The values for both the datasets of all three genomes is tabulated in Table 2.

**Table 2:** Statistics of alternative splicing events for all AS genes and the orthologous AS gene subset (gene level analysis)

Type of alternative splicing event	Bovine (Complete set)	Bovine (Orthologous set)	Human (Complete set)	Human (Orthologous set)	Mouse (Complete set)	Mouse (Orthologous set)
Transcriptional Start Site	3136 (69%)	2380 (68%)	16180 (97%)	3771 (98%)	15357 (93%)	3629 (96%)
Alternative Initiation Exons	2476 (54%)	1795 (51%)	13605 (81%)	3379 (88%)	12142 (74%)	3117 (82%)
Transcriptional Termination Site	3787 (83%)	2358 (67%)	16558 (99%)	3778 (98%)	16111 (98%)	3621 (96%)
Alternative Termination Exons	3306 (72%)	1774 (51%)	15060 (90%)	3401 (89%)	14276 (86%)	3136 (83%)
Alternative Acceptor	404 (9%)	344 (10%)	4580 (27%)	1270 (33%)	3265 (20%)	926 (25%)
Alternative Donor	454 (10%)	394 (11%)	4517 (27%)	1209 (32%)	3297 (20%)	925 (25%)
Cassette Exons	2706 (59%)	2227 (64%)	10135 (60%)	2757 (72%)	7081 (43%)	2120 (56%)
Intron Retention	875 (19%)	695 (20%)	5564 (33%)	1468 (38%)	4348 (26%)	1153 (31%)
Mutually Exclusive	148 (3%)	124 (4%)	382 (3%)	158 (4%)	158 (1%)	70 (2%)

Data on nine AS events described in Table 4 have been compiled.

**Figure 5:**
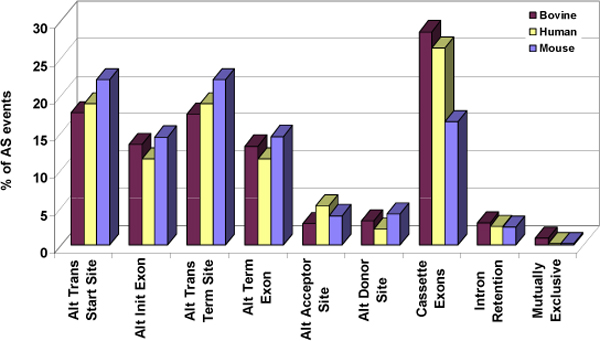
**Distribution of alternative splicing events-event level analysis for bovine, human and mouse orthologous AS genes**. Event level analysis of each of the nine events, described in Figure 1 and based on the data in Table 3.

It should be noted that each AS gene contains several events. The distribution of each event compared to the total number of AS events observed in the orthologous set of the three genomes represent the event level analysis as shown in Equation 2. (Table 3, Figure 5).

**Table 3:** Statistics of alternative splicing events for the orthologous gene subset (event level analysis)

Type of alternative splicing event	Bovine	Human	Mouse
Transcriptional Start Site	5177 (17%)	28311 (19%)	21556 (22%)
Alternative Initiation Exons	3923 (13%)	17177 (11%)	14030 (14%)
Transcriptional Termination Site	5126 (17%)	28264 (19%)	21534 (22%)
Alternative Termination Exons	3851 (13%)	17243 (11%)	14119 (14%)
Alternative Acceptor	822 (3%)	7790 (5%)	3820 (4%)
Alternative Donor	934 (3%)	7582 (2%)	3964 (4%)
Cassette Exons	8337 (28%)	39368 (26%)	15943 (16%)
Intron Retention	853 (3%)	3694 (3%)	2307 (2%)
Mutually Exclusive	254 (1%)	344 (0.2%)	155 (0.2%)
**Total events**	**29277**	**149773**	**97428**

Data on nine AS events described in Table 4 have been compiled.

Considerable conservation was observed in each of the nine AS events for the three species. Our analysis proves that exon skipping or cassette exon is the most prevalent internal AS event in the orthologous genes of all three species, comprising 28%, 26% and 16% of all AS events in bovine, human and mouse, respectively. On the other hand, intron retention and mutually exclusive exons were the least favoured AS events. Intron retention accounted for only 3% of bovine AS events, compared to 3% in human and 2% in mouse. Haussler and co-workers [35] estimated 38% exon skipping and 3% intron retention in human, which are very similar to our values. ASD [3,4] reports 52% cassette exons and 17% intron retention, which differ considerably from our calculations. This could however be due to the fact that ASD has used the entire human genome for their calculations whereas we have only utilized orthologous AS genes for our analysis.

Overall, from the two sets of analyses, fewer bovine genes show equivalent % of AS events compared to human and mouse, which implies that these orthologous AS genes in cow show high variation between the transcripts structure, despite low number of actually different transcripts as opposed to human and mouse genes.

**Figure 6:**
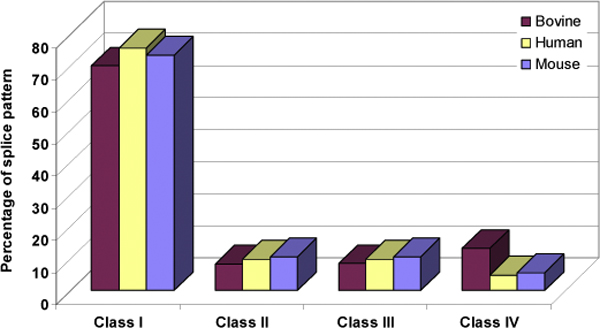
**Splicing pattern distribution in the orthologous bovine, human and mouse alternatively spliced genes**. Statistics on four component splicing patterns have been complied, with the transcript diversity index defined as the fraction of all patterns involving variant exons.

The splicing pattern analysis was done for the orthologous AS genes by calculating the percentage of the four classes in the splicing pattern to determine the exact nature of the transcript diversity. Among all the patterns described above we observe that Class I (Distinct-Distinct) patterns have the highest occurrence (70%) (Table 4 and Figure 6). Class IV (Variable-Variable) is over-represented (13%) in bovine genes compared to human (5%) and mouse (6%). The diversity in bovine AS genes is thus predominantly composed of edges linking two variable exons, as opposed to human and mouse AS genes, where the transcript diversity is predominantly composed of edges linking a distinct exon with a variable one or *vice versa*.

**Table 4:** Alternative splicing class distribution based on splicing patterns for orthologous bovine, human and mouse AS genes

Genome	Class I	Class II	Class III	Class IV
Bovine	73588 (70%)	8776 (8%)	8843 (8%)	13754 (13%)
Human	223022 (75%)	28725 (10%)	28759 (10%)	14299 (5%)
Mouse	149036 (73%)	21281 (10%)	21284 (10%)	11298 (6%)

### Splice site motif analysis

The splice site motif analysis yielded consistent values in the three genomes. 99% of the splice site motifs in bovine AS genes were found to be GT-AG (Table 5). The data for the orthologous AS gene set is very similar (data not shown).

**Table 5:** Splice site motif analysis for bovine, human and mouse AS genes

Splice site motifs	Bovine	Human (ASTD)	Mouse (ASTD)
**GT-AG**	99%	98%	99%
**GC-AG**	0.93%	2%	1%
**AT-AC**	0.07%	0%	0%

### GO analysis of orthologous gene sets

**Figure 7:**
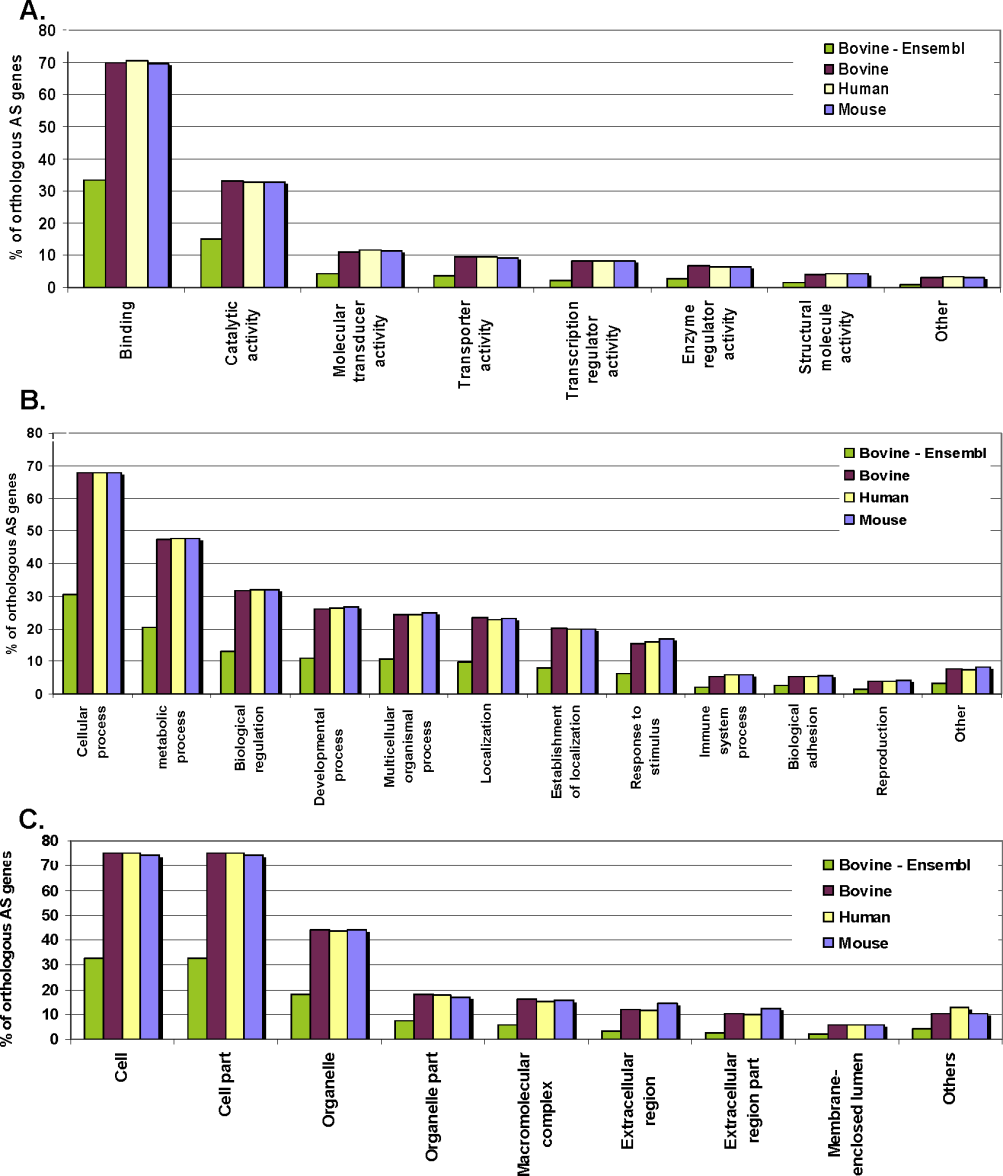
**Occurrence of gene ontology (GO) terms in bovine, human and mouse for the orthologous AS gene subset**. GO terms have been categorized on the basis of A. molecular function, B. biological process and C. cellular component.

Gene Ontology (GO) analysis was carried out for all three organisms on the orthologous AS gene set where the GO categories were selected based on the work done by Chen *et al *[33]. The transcript sequences for the orthologous AS genes of human, mouse and bovine were analyzed. It was observed that the overall GO categories for all the three species were very similar (Table 6 and Figure 7). In the area of molecular function, the highest functionality was observed for "binding" in all three species. In terms of biological process, "cellular processes" was the preferred category, while for cellular component, "cell part" was most popular. This high similarity in functionality could reflect the common lineage of bovine, human and mouse, as mammalian.

**Table 6:** Gene ontology (GO) annotation summary for the orthologous AS gene set.

A. Molecular function	%Bovine (Ensembl)	% Bovine genes	% Human genes	% Mouse Genes
Binding	33.5	69.86	70.43	69.53
Catalytic activity	14.9	33.22	32.67	32.8
Molecular transducer activity	4.42	11.07	11.58	11.47
Transporter activity	3.54	9.56	9.41	9.33
Transcription regulator activity	2.03	8.33	8.16	8.35
Enzyme regulator activity	2.8	6.68	6.39	6.44
Structural molecule activity	1.57	4.11	4.28	4.32
Other	0.94	3.08	3.34	3.07

**B. Biological process**	**% Bovine** **(Ensembl)**	**% Bovine** **genes**	**% Human** **genes**	**% Mouse** **Genes**

Cellular process	30.62	67.78	67.77	67.97
metabolic process	20.58	47.26	47.67	47.72
Biological regulation	12.99	31.74	31.89	31.96
Developmental process	10.9	26.08	26.44	26.79
Multicellular organismal process	10.53	24.2	24.35	24.75
Localization	9.67	23.43	22.69	23.21
Establishment of localization	8.08	20.12	19.74	19.82
Response to stimulus	6.22	15.33	16.01	16.93
Immune system process	2.14	5.25	5.84	5.88
Biological adhesion	2.63	5.37	5.35	5.59
Reproduction	1.51	3.82	3.96	4.03
Other	3.14	7.85	7.46	8.29

**C. Cellular component**	**%Bovine** **(Ensembl)**	**% Bovine** **genes**	**% Human** **genes**	**% Mouse** **Genes**

Cell	32.65	74.89	75.05	74.3
Cell part	32.65	74.89	75.05	74.3
Organelle	18.21	44.12	43.75	44.01
Organelle part	7.31	17.98	17.55	17.1
Macromolecular complex	5.88	15.95	15.33	15.55
Extracellular region	3.37	11.84	11.45	14.38
Extracellular region part	2.28	10.45	10.09	12.35
Membrane-enclosed lumen	1.86	5.76	5.63	5.88
Others	4.17	10.25	12.7	10.2

However, a similar plot was also created for the bovine genome, using a different set of annotations, where the entire GO details were obtained from Ensembl using the BioMart tool [28]. This analysis showed considerably low percentage for bovine as opposed to the previous plot. This, we believe can be a result of low level of annotation available for bovine genes. In this plot, a considerable drop in functionality was noticed across all the areas for bovine genome (Table 6 and Figure 7). Therefore, we were able to identify 38% more functional information in terms of GO annotations than currently available in Ensembl for bovine genes.

### Mapping of human disease genes to bovine orthologous genes

The use of farm animals like cattle, pigs, sheep, goats, horses and chickens as research models has won many Nobel Prizes for researchers worldwide [36]. Various new opportunities in areas of biomedical research have been created by the application of the tools for genetic manipulation and genomic sequencing in farm animals [16]. This provides valuable insights into gene function and genetic and environmental influences on animal production and human diseases [36]. Because of the size and relatively long intervals between generations, domestic species are widely used to unravel the mechanisms involved in programming the development of an embryo and fetus, resulting in adult onset of diseases [37,38]. Rogers *et al*. [39] have identified that the CFTR gene knockout model of pig better mimics human pathology than mouse models as they fail to develop the hallmark pancreatic, lung and intestinal obstructions that occur in humans. Reynolds *et al*. [40] note that surgery, blood sampling, tissue recovery, serial biopsies, instrumentations, whole organ manipulations and many other biomedical applications are more easily achieved in animals larger than a mouse, suggesting that size does matter when it comes to animal models. Hence mapping human disease genes to bovine orthologous genes is an excellent mode for carrying out analytical work and verifying the suitability of cow as a model organism.

Out of the 94 human disease genes that were collected, we observed splicing in 66 cases, (70.21%). Mapping these 66 spliced human genes onto orthologous bovine genes suggested that only 17 of the orthologous bovine genes were spliced (18.09%). Cassette exons occur in 38 of human disease genes (120 cassette exons, Table 7) and 14 orthologous bovine genes. At the exon level, we observed that 97 of 120 human exons (Table 7) were conserved in bovine, indicating a high level of conservation in this dataset across both the species. Previously, for a larger dataset [16], it was reported that majority of genes with cassette exons in human were present and regulated in cattle. However, at the gene level, for the current dataset, we have observed that only 3 genes with cassette exons in human (Table 8) were present and regulated in bovine.

**Table 7:** Human disease genes: Conservation of cassette exons in bovine orthologous genes.

Number of cassette exons in 38 AS human disease genes	120
Exons present and constitutive in bovine orthologous gene	90
Exons present and regulated in bovine orthologous gene	7
Exons absent in bovine orthologous gene	23

**Table 8:** Human disease genes: Cassette exons present and regulated in bovine orthologous genes.

Disease	Gene name	Ensembl human transcript ID	Cassette exon position in human transcript	Ensembl bovine transcript ID	Cassette exon position in bovine orthologous transcript
**Colon Cancer**	MLH1	ENST00000231790	Exon9	ENSBTAT00000022288	Exon9
			Exon10		Exon10
**Spinal muscular atrophy**	SMN1	ENSG00000172062	Exon6	ENSBTAT00000007547	Exon6
			Exon5		Exon5
			Exon32		Exon32
**Tangier disease**	ABC1	ENST00000341579	Exon2	ENSBTAT00000027538	Exon2
			Exon10		Exon10

We also carried out a detailed survey of the 94 human disease genes to identify intron retention events. We noted intron retention in nine human genes out of which, in five genes IR was present and constitutive in bovine (> 50%; Table 9). It has been indicated before that the expression of intron-containing sequences occur in a variety of diseases [41].

**Table 9:** Human disease genes: Intron retention present and constitutive in bovine orthologous genes.

Diseases	Gene Name	Transcript ID	Intron Ret	Transcript ID	Conserved exon in bovine
**Glaucoma**	GLC1A	ENST00000037502	Exon1	ENSBTAT00000027111	Exon1
**Spinocerebellar ataxia**	SCA1	ENST00000244769	Exon9	ENSBTAT00000044692	Exon3
**Polycystic kidney disease**	PKD1	ENST00000262304	Exon23	ENSBTAT00000027480	Exon23
			Exon 15		Exon15
**Autoimmune polyglandular syndrome**	APS1	ENST00000291582	Exon10	ENSBTAT00000031852	Exon10
**Wilson's disease**	ATP7B	ENST00000242839	Exon2	ENSBTAT00000013674	Exon1

### Protein domain analysis of the orthologous disease gene set

For the eight human disease genes that have orthologous genes in the bovine genome, (three genes with CE and five genes with IR), protein domain analysis revealed that AS affects the structure and function of the proteins encoded by the various transcripts from these genes. It was evident that due to AS, the majority of the transcripts either lacked the complete functional domain or lacked an essential component/segment of the functional domain. This suggests that AS is a major machanism that could render these proteins non-functional, besides perturbing the structure or fold of the protein.

For the set of the bovine orthologous genes, only two of eight genes appear to be spliced, resulting in probable structure and function disruption. These genes are responsible for spinal muscular atrophy and colon cancer, with the former noted as a disease caused by AS [1]. Further investigation revealed that four of these eight genes had all the domains from their human counterparts conserved. This implies that 4/8 orthologous bovine genes (including the two AS genes) had essential segments or complete functional domains missing, due to AS.

Wilson's disease is another disease that has been characterised in cow (OMIA). We observe that the human gene known to be responsible for this disease has a retained intron in one of its transcripts, which is orthologous to the only transcript available in the corresponding bovine gene. Thus, the cow would be most suitable as a model organism for this human disease.

## Conclusion

This is the first comprehensive study of the bovine transcriptome, with 21% of bovine genes exhibiting alternative splicing, compared to 68% and 57% in human and mouse, respectively. Our analyses show that bovine AS genes are composed of fewer transcripts but many more exons than human and mouse AS genes, although comprising exons and introns of comparable extents. Nine different splicing events were compared among cow, human and mouse genomes. Compared to their human and mouse counterparts many more bovine AS genes show intron retention. The most common AS event was found to be exon skipping and the least common events were intron retention and mutually exclusive exons. With predominantly introns linking two variable exons, as opposed to human and mouse genes fewer AS bovine genes show high transcript variability.

38% more functional information than currently available in Ensembl was identified with our approach which helped us collate the GO annotations for bovine AS genes. The orthologous bovine AS genes are functionally very similar to human and mouse genes as suggested by GO annotations.

From the results of our protein domain analysis it is evident that AS plays a major role in disease implications in both human and cow, and is suitable as a model for investigating spinal muscular atrophy, colon cancer, tangier disease, glaucoma, spinocerebellar ataxia, polycystic kidney disease, autoimmune poly grandular syndrome and wilson's disease. Our results provide a window of opportunity for more in-depth analysis over a larger dataset, where the cow can serve as a model organism for many more human diseases.

## Competing interests

The authors declare that they have no competing interests.

## Authors' contributions

SR conceived the alternative splicing analysis concept for the bovine genome. EC obtained the data and carried out the analysis. EC and SR wrote the paper. All authors approved the manuscript and declare that there is no conflict of interest.

## Note

Other papers from the meeting have been published as part of *BMC Bioinformatics *Volume 10 Supplement 15, 2009: Eighth International Conference on Bioinformatics (InCoB2009): Bioinformatics, available online at http://www.biomedcentral.com/1471-2105/10?issue=S15.

## References

[B1] Caceres JF Komblihtt AR Alternative Splicing: multiple control mechanisms and involvement in human disease Trends in Genetics 2002 18 186 193 10.1016/S0168-9525(01)02626-9 11932019

[B2] Tazi J Bakkour N Stamm S Alternative splicing and disease Biochimica et Biophysica Acta 2009 14 26 18992329 10.1016/j.bbadis.2008.09.017 PMC5632948

[B3] Thanaraj TA Stamm S Clark F Riethoven JJ Le TV Muilu J ASD: the Alternative Splicing Database Nucleic Acids Res 2004 32 Database issue D64 D69 10.1093/nar/gkh030 14681360 PMC308764

[B4] Stamm S Riethoven JJ Le Texier V Gopalakrishnan C Kumanduri V Tang Y Barbosa-Morais NL Thanaraj TA ASD: a bioinformatics resource on alternative splicing Nucleic Acids Res 2006 34 Database issue D46 D55 10.1093/nar/gkj031 16381912 PMC1347394

[B5] Kim N Alekseyenko AV Roy M Lee C The ASAP II database: analysis and comparative genomics of alternative splicing in 15 animal species Nucleic Acids Res 2007 35 D93 D98 10.1093/nar/gkl884 17108355 PMC1669709

[B6] Lee BTK Tan TW Ranganathan S DEDB: a database of Drosophila melanogaster exons in splicing graph form BMC Bioinformatics 2004 5 189 10.1186/1471-2105-5-189 15581431 PMC538278

[B7] Leipzig J Pevzner P Heber S The Alternative Splicing Gallery (ASG): bridging the gap between genome and transcriptome Nucleic Acids Res 2004 32 3977 3983 10.1093/nar/gkh731 15292448 PMC506815

[B8] Bollina D Lee BTK Tan TW Ranganathan S ASGS: an alternative splicing graph web service Nucleic Acids Res 2006 34 W444 W447 10.1093/nar/gkl268 16845045 PMC1538904

[B9] Lee Y Lee Y Kim B Shin Y Nam S Kim P Kim N Chung WH Kim J Lee S ECgene: an alternative splicing database update Nucleic Acids Res 2006 35 D99 D103 10.1093/nar/gkl992 17132829 PMC1716719

[B10] Krawczak M Reiss J Cooper DN The mutational spectrum of single base-pair substitutions in mRNA splice junctions of human genes: causes and consequences Hum Genet 1992 90 1-2 41 54 10.1007/BF00210743 1427786

[B11] Lee C Wang Q Bioinformatics analysis of alternative splicing Brief Bioinform 2005 6 23 33 10.1093/bib/6.1.23 15826354

[B12] Brett D Pospisil H Valcarcel J Reich J Bork P Alternative splicing and genome complexity Nature Genet 2001 30 29 30 10.1038/ng803 11743582

[B13] Huber W Carey VJ Long L Falcon S Gentleman R Graphs in molecular biology BMC Bioinformatics 2007 8 Suppl 6 S8 10.1186/1471-2105-8-S6-S8 17903289 PMC1995545

[B14] Modrek B Lee C A genomic view of alternative splicing Nature Genet 2002 30 13 19 10.1038/ng0102-13 11753382

[B15] Heber S Alekseyev M Sze SH Tang H Pevzner PA Splicing graphs and EST assembly problem Bioinformatics 2002 18 S181 S188 12169546 10.1093/bioinformatics/18.suppl_1.s181

[B16] The Bovine Genome Sequencing Consortium Elsik CG Tellam RL Worley KC Gibbs RA Muzny DM Weinstock GM Adelson DL Eichler EE Elnitski L Guigó R Hamernik DL Kappes SM Lewin HA Lynn DJ Nicholas FW Reymond A Rijnkels M Skow LC Zdobnov EM Schook L Womack J Alioto T Antonarakis SE Astashyn A Chapple CE Chen HC Chrast J Câmara F Ermolaeva O Henrichsen CN Hlavina W Kapustin Y Kiryutin B Kitts P Kokocinski F Landrum M Maglott D Pruitt K Sapojnikov V Searle SM Solovyev V Souvorov A Ucla C Wyss C Anzola JM Gerlach D Elhaik E Graur D Reese JT Edgar RC McEwan JC Payne GM Raison JM Junier T Kriventseva EV Eyras E Plass M Donthu R Larkin DM Reecy J Yang MQ Chen L Cheng Z Chitko-McKown CG Liu GE Matukumalli LK Song J Zhu B Bradley DG Brinkman FS Lau LP Whiteside MD Walker A Wheeler TT Casey T German JB Lemay DG Maqbool NJ Molenaar AJ Seo S Stothard P Baldwin CL Baxter R Brinkmeyer-Langford CL Brown WC Childers CP Connelley T Ellis SA Fritz K Glass EJ Herzig CT Iivanainen A Lahmers KK Bennett AK Dickens CM Gilbert JG Hagen DE Salih H Aerts J Caetano AR Dalrymple B Garcia JF Gill CA Hiendleder SG Memili E Spurlock D Williams JL Alexander L Brownstein MJ Guan L Holt RA Jones SJ Marra MA Moore R Moore SS Roberts A Taniguchi M Waterman RC Chacko J Chandrabose MM Cree A Dao MD Dinh HH Gabisi RA Hines S Hume J Jhangiani SN Joshi V Kovar CL Lewis LR Liu YS Lopez J Morgan MB Nguyen NB Okwuonu GO Ruiz SJ Santibanez J Wright RA Buhay C Ding Y Dugan-Rocha S Herdandez J Holder M Sabo A Egan A Goodell J Wilczek-Boney K Fowler GR Hitchens ME Lozado RJ Moen C Steffen D Warren JT Zhang J Chiu R Schein JE Durbin KJ Havlak P Jiang H Liu Y Qin X Ren Y Shen Y Song H Bell SN Davis C Johnson AJ Lee S Nazareth LV Patel BM Pu LL Vattathil S Williams RL Jr Curry S Hamilton C Sodergren E Wheeler DA Barris W Bennett GL Eggen A Green RD Harhay GP Hobbs M Jann O Keele JW Kent MP Lien S McKay SD McWilliam S Ratnakumar A Schnabel RD Smith T Snelling WM Sonstegard TS Stone RT Sugimoto Y Takasuga A Taylor JF Van Tassell CP Macneil MD Abatepaulo AR Abbey CA Ahola V Almeida IG Amadio AF Anatriello E Bahadue SM Biase FH Boldt CR Carroll JA Carvalho WA Cervelatti EP Chacko E Chapin JE Cheng Y Choi J Colley AJ de Campos TA De Donato M Santos IK de Oliveira CJ Deobald H Devinoy E Donohue KE Dovc P Eberlein A Fitzsimmons CJ Franzin AM Garcia GR Genini S Gladney CJ Grant JR Greaser ML Green JA Hadsell DL Hakimov HA Halgren R Harrow JL Hart EA Hastings N Hernandez M Hu ZL Ingham A Iso-Touru T Jamis C Jensen K Kapetis D Kerr T Khalil SS Khatib H Kolbehdari D Kumar CG Kumar D Leach R Lee JC Li C Logan KM Malinverni R Marques E Martin WF Martins NF Maruyama SR Mazza R McLean KL Medrano JF Moreno BT Moré DD Muntean CT Nandakumar HP Nogueira MF Olsaker I Pant SD Panzitta F Pastor RC Poli MA Poslusny N Rachagani S Ranganathan S Razpet A Riggs PK Rincon G Rodriguez-Osorio N Rodriguez-Zas SL Romero NE Rosenwald A Sando L Schmutz SM Shen L Sherman L Southey BR Lutzow YS Sweedler JV Tammen I Telugu BP Urbanski JM Utsunomiya YT Verschoor CP Waardenberg AJ Wang Z Ward R Weikard R Welsh TH Jr White SN Wilming LG Wunderlich KR Yang J Zhao FQ The Genome Sequence of Taurine Cattle: A Window to Ruminant Biology and Evolution Science 324 522 528 10.1126/science.1169588 19390049 PMC2943200

[B17] Miziara1 MN Riggs PK Amaral MEJ Comparative analysis of noncoding sequences of orthologous bovine and human gene pairs Genetics and Molecular Research 2004 3 465 473 15688313

[B18] Gibbs R Weinstock G Bovine Genome Sequencing Initiative - Cattle-izing the Human Genome http://www.genome.gov/Pages/Research/Sequencing/SeqProposals/BovineSEQ.pdf accessed on 15/05/2009.

[B19] Lewin HA It's a Bull's Market Science 2009 324 478 479 19390037 10.1126/science.1173880

[B20] Ashburner M Ball CA Blake JA Botstein D Butler H Cherry JM Davis AP Dolinski K Dwight SS Eppig JT Harris MA Hill DP Issel-Tarver L Kasarskis A Lewis S Matese JC Richardson JE Ringwald M Rubin GM Sherlock G Gene ontology: tool for the unification of biology. The Gene Ontology Consortium Nat Genet 2000 25 25 29 10.1038/75556 10802651 PMC3037419

[B21] NCBI Genes and Disease database http://www.ncbi.nlm.nih.gov/books/bv.fcgi?rid=gnd accessed on 6/5/2009.

[B22] Hubbard TJ Aken BL Beal K Ballester B Caccamo M Chen Y Clarke L Coates G Cunningham F Cutts T Down T Dyer SC Fitzgerald S Fernandez-Banet J Graf S Haider S Hammond M Herrero J Holland R Howe K Howe K Johnson N Kahari A Keefe D Kokocinski F Kulesha E Lawson D Longden I Melsopp C Megy K Meidl P Ouverdin B Parker A Prlic A Rice S Rios D Schuster M Sealy I Severin J Slater G Smedley D Spudich G Trevanion S Vilella A Vogel J White S Wood M Cox T Curwen V Durbin R Fernandez-Suarez XM Flicek P Kasprzyk A Proctor G Searle S Smith J Ureta-Vidal A Birney E Ensembl 2007 Nucleic Acids Res 2007 35 D610 D617 10.1093/nar/gkl996 17148474 PMC1761443

[B23] GTF: an Exchange Format for Feature Description http://www.sanger.ac.uk/Software/formats/GFF/accessed on 08/09/2008.

[B24] Chacko E Ranganathan S Comprehensive splicing graph analysis of alternative splicing patterns in chicken, compared to human and mouse BMC Genomics 2009 10 Suppl 1 S5 10.1186/1471-2164-10-S1-S5 19594882 PMC2709266

[B25] Iseli C Jongeneel CV Bucher P ESTScan: a program for detecting, evaluating, and reconstructing potential coding regions in EST sequences Proc Int Conf Intell Syst Mol Biol 1999 138 148 10786296

[B26] Conesa A Gotz A Garcia-Gomez JM Terol J Talon M Robles M Blast2Go: a universal tool for annotation, visualization and analysis in functional genomics research Bioinformatics 2005 21 3674 3676 10.1093/bioinformatics/bti610 16081474

[B27] Nagaraj SH Deshpande N Gasser RB Ranganathan S ESTExplorer: an expressed sequence tag (EST) assembly and annotation platform Nucleic Acids Research 2007 35 W143 W147 10.1093/nar/gkm378 17545197 PMC1933243

[B28] Ye J Fang L Zheng H Zhang Y Chen J Zhang Z Wang J Li S Li R Bolund L Wang J WEGO: a tool for plotting GO annotations Nucleic Acids Res 2006 34 Web Server issue W293 W297 10.1093/nar/gkl031 16845012 PMC1538768

[B29] Durinck S Moreau Y Kasprzyk A Davis S De Moor B Brazma A Huber W BioMart and Bioconductor: a powerful link between biological databases and microarray data analysis Bioinformatics 2005 21 3439 40 10.1093/bioinformatics/bti525 16082012

[B30] Thompson JD Gibson TJ Plewniak F Jeanmougin F Higgins DG The CLUSTAL_X windows interface: flexible strategies for multiple sequence alignment aided by quality analysis tools Nucleic Acids Research 1997 25 4876 4882 10.1093/nar/25.24.4876 9396791 PMC147148

[B31] Finn RD Tate J Mistry J Coggill PC Sammut JS Hotz HR Ceric G Forslund K Eddy SR Sonnhammer EL Bateman A The Pfam protein families database Nucleic Acids Research 2008 36 D281 D288 10.1093/nar/gkm960 18039703 PMC2238907

[B32] Nagasaki H Arita M Nishizawa T Suwa M Gotoh O Species-specific variation of alternative spicing and transcriptional initiation in six eukaryotes Gene 2005 364 53 62 10.1016/j.gene.2005.07.027 16219431

[B33] Chen FC Chen CJ Ho JY Chuang TJ Identification and evolutionary analysis of novel exons and alternative splicing events using cross-species EST-to-genome comparisons in human, mouse and rat BMC Bioinformatics 2006 7 136 10.1186/1471-2105-7-136 16536879 PMC1479377

[B34] Deutsch M Long M Intron-exon structures of eukaryotic model organisms Nucleic Acids Res 1999 27 3219 3228 10.1093/nar/27.15.3219 10454621 PMC148551

[B35] Sugnet CW Kent WJ Ares M Jr Haussler D Transcriptome and genome conservation of alternative splicing events in humans and mice Pac Symp Biocomput 2004 66 77 14992493 10.1142/9789812704856_0007

[B36] Roberts RM Smith GW Bazer FW Cibelli J Seidel GE Jr Bauman DE Reynolds LP Ireland JJ Farm animal research in crisis Science 2009 324 468 469 10.1126/science.1168521 19390030

[B37] King AJ Olivier NB Mohankumar PS Lee JS Padmanabhan V Fink GD Hypertension caused by prenatal testosterone excess in female sheep American journal of physiology. Endocrinology and metabolism 2007 292 E1837 10.1152/ajpendo.00668.2006 17327368

[B38] Padmanabhan V Environment and origin of disease Rev Endocr Metab Disord 2007 8 67 69 10.1007/s11154-007-9051-3 17712637

[B39] Rogers CS Stoltz DA Meyerholz DK Ostedgaard LS Rokhlina T Taft PJ Rogan MP Pezzulo AA Karp PH Itani OA Kabel AC Wohlford-Lenane CL Davis GJ Hanfland RA Smith TL Samuel M Wax D Murphy CN Rieke A Whitworth K Uc A Starner TD Brogden KA Shilyansky J McCray PB Jr Zabner J Prather RS Welsh MJ Disruption of the CFTR gene produces a model of cystic fibrosis in newborn pigs Science 2008 321 1837 18818360 10.1126/science.1163600 PMC2570747

[B40] Reynolds LP Ireland JJ Caton JS Bauman DE Davis TA Commentary on domestic animals in agricultural and biomedical research: an endangered enterprise Journal of Nutrition 2009 139 427 10.3945/jn.108.103564 19158219 PMC3314500

[B41] Forrest ST Barringhaus KG Perlegas D Hammarskjold ML McNamara CA Intron Retention Generates a Novel Id3 Isoform That Inhibits Vascular Lesion Formation The Journal of biological chemistry 2004 279 32897 32903 10.1074/jbc.M404882200 15159391

